# Focus on the target: the tumor microenvironment, Society for Immunotherapy of Cancer Annual Meeting Workshop, October 24th-25th 2012

**DOI:** 10.1186/2051-1426-1-9

**Published:** 2013-06-27

**Authors:** Melek Erdinc Sunay, Francesco Marincola, Samir N Khleif, Samuel C Silverstein, Bernard A Fox, Jerome Galon, Leisha A Emens

**Affiliations:** 1 Oncology Department, Johns Hopkins University, 1650 Orleans St., CRB1 Room 484, Baltimore, MD 21287, USA; 2 Sidra Medical and Research Center, Sidra Tower, AL Corniche Street, Qatar Foundation, P.O. Box 26999, Doha, Qatar; 3 Georgia Health Sciences Cancer Center Director, Georgia Regents University, 1411 Laney Walker Blvd, Augusta, GA 30912, USA; 4 Physiology and Cellular Biophysics, Columbia University, 630 W 168 Street (P&S 11-444), New York, NY 10032, USA; 5 College of Physicians and Surgeons, 630 W 168 Street (P&S 11-444), New York, NY 10032, USA; 6 Earle A. Chiles Research Institute, Molecular and Tumor Immunology Lab, 4805 NE Glisan, 2N35, Portland, OR 97213, USA; 7 INSERM- Cordeliers Research Center, Integrative Cancer Immunology Team, 15 Rue De L Ecole De Medecine, Paris 75006, France; 8 Johns Hopkins University, 1650 Orleans Street, Rm.409, Cancer Research Building 1, Baltimore, MD 21231-1000, USA

**Keywords:** Tumor microenvironment, Immunotherapy, Cancer, Tumor immunology, Suppressor cells, Immunoscore

## Abstract

The Workshop associated with the 27th Annual Meeting of the Society for Immunotherapy of Cancer (SITC), North Bethesda, MD, October 24-25, 2012 focused on targeting the tumor microenvironment as part of an integrative approach to immune-based cancer therapy.

## 

Established tumors are complex, heterogeneous masses composed of malignant cells admixed with a variety of non-transformed host cells, including stromal cells, endothelial cells, and immune cells. This elaborate web of diverse cell types fosters malignant tumor cell interactions with the tumor associated vasculature and fibroblasts as well as a variety of immune cells in support of tumor growth, invasion, and metastasis. The importance of these dynamic cellular interactions in the development of cancer has stimulated strong interest in evaluating components of the tumor microenvironment as targets for cancer therapy. Multiple lines of research aim to dissect the tumor microenvironment to gain insight into cancer prognosis and treatment selection, as well as to further understand the mechanisms that drive immune-based tumor rejection.

To support these efforts, the Society for Immunotherapy of Cancer (SITC) held a workshop focused on targeting the tumor microenvironment as part of an integrative approach to immune-based cancer therapy. SITC is a non-profit group of medical professionals established in 1984 to facilitate the exchange and promotion of scientific information about the promise and breakthroughs of immunotherapy for cancer patients. Society members include a constituency of nearly 600 clinical and basic scientists from around the world working in academia, industry, and governmental regulatory agencies. SITC’s members represent 17 medical specialties and are engaged in research and treatment of most types of cancer. The Society was founded on the belief that new immune-based treatments would continue to complement traditional cancer treatments and move into the mainstream in the fight against cancer. To aid in this effort, SITC provides a venue to facilitate the discussion of current clinical trial results and methodologies, as well as means to collaborate on new initiatives in tumor immunology and cancer immunotherapy with the ultimate goal of improving cancer patient outcomes.

The following themes were selected for the focus of the 2012 SITC workshop: (1) cellular and molecular interactions within the tumor microenvironment that impact the activities of innate and antigen-specific immune cells; (2) manipulation of these interactions to remodel the tumor microenvironment and promote tumor regression (3) current and future combination cancer immunotherapies for clinical use that actively target components of the tumor microenvironment to provoke lasting tumor immunity and improve patient outcomes; and (4) the tumor Immunoscore as a new indicator of prognosis, and predictive marker of response to cancer therapy.

## Immunoregulatory components of the tumor microenvironment

The host antitumor immune response can sculpt tumor growth, invasion, and metastasis in a variety of ways. The prevention of immune cell access into the tumor, the accumulation of inhibitory FoxP3^+^ regulatory T cells (Treg) and/or myeloid-derived suppressor cells (MDSCs), the activation of negative immunoregulatory pathways, and the dysregulation of effector T cells are all mechanisms by which tumors evade the host immune system. Notably, the presence of large numbers of tumor infiltrating T lymphocytes (TILs) has been reported to be an indicator of good prognosis in multiple solid tumors [1-5]. Therefore, it is not surprising that physically preventing effector CD8^+^ T cell infiltration or inhibiting their activity once they gain access to the tumor might be a means by which tumors protect themselves from immune attack, enabling them to persist within the host.

Dr. George Coukos presented an elegant characterization of the immunobiology of ovarian cancers. Almost 50% of ovarian cancer patients lack CD3^+^ TILs within nests of tumor epithelial cells, despite the presence of CD3^+^ TILs within the host stroma [4]. The absence of intratumoral TILs is correlated with decreased survival in these patients. Tumor endothelial cells (TECs) present at the blood-tumor barrier act as gatekeepers, regulating the homing, adhesion and transendothelial migration of lymphocytes into the tumor [6]. Dr. Coukos and his team further studied the dynamic interactions between tumor cells, endothelial cells and T cells in ovarian cancer. Analysis of ovarian tumors by immuohistochemistry (IHC) detected an overexpression of endothelin type B receptor (ETBR) by the tumor-associated vasculature and stromal cells within ovarian tumors with decreased CD3^+^ TIL. ETBR expression was associated with the loss of T cell infiltration into tumors and down regulation of Intercellular Adhesion Molecule-1 (ICAM-1 or CD54) expression, a T cell adhesion molecule expressed by endothelial cells. In an ID8 ovarian cancer and a human papillomavirus E6/E7 transformed model (TC-1), blockade of ETBR enhanced T cell infiltration and augmented vaccine-induced tumor regression without an increase in systemic T cell number or activity [6]. In addition, many TECs express Fas-ligand (FasL or CD95L), and induce the death of Fas-expressing T cells attempting to gain access to the tumor. Thus, TECs can create a protective barrier to block or disrupt transendothelial T cell migration and survival within the tumor microenvironment [6].

If high levels of TILs can be associated with better progression-free and overall survival, then why does the presence of TILs not always result in tumor rejection? Part of the reason is that both innate and adaptive immune cells that gain access to the tumor site can contribute to disease progression. They do this by corrupting the inherent protective inflammatory response mounted against the tumor to promote immune evasion. Alterations in tumor cell biology can lead to decreased susceptibility to killing, and alterations in antigen presenting cells can lead to faulty T cell priming and promote T cell dysfunction. Both the induction of suppressive cytokines and the expression of negative immunomodulatory molecules within the tumor microenvironment can dampen immune responses. High levels of interleukin-10 (IL-10) and/or transforming growth factor-β (TGF-β), the expression of FAS ligand (FASL), programmed cell death ligand-1 (PDL-1 or CD274) and programmed cell death ligand-2 (PDL-2), and the expression of immunomodulatory enzymes like indoleamine 2,3-dioxygenase, (IDO), arginase (ARG) or inducible nitric-oxide synthase (iNOS) can inhibit tumor immunity [7]. The major producers of these immunoregulatory molecules include toleragenic dendritic cells (DCs), Tregs, MDSCs, and tumor-associated macrophages (TAMs).

Dr. Hans Schreiber discussed the role of stromal cells in cross-presenting tumor antigens as a means of protecting from cancer relapse. In particular, antigen loss variant-tumor cells can be eliminated as bystanders as a secondary effect of destruction of the stroma that supports them, provided adequate levels of tumor antigen are available for stromal cross-presentation [8]. Furthermore, treating with radiation or chemotherapy releases sufficient quantities of tumor antigen to sensitize stromal cells for destruction by CD8^+^ cytotoxic T lymphocytes [9].

Dr. David Munn discussed metabolic immunoregulatory pathways that promote suppression in the tumor microenvironment. One example is the IDO pathway. Within the tumor stroma, IDO is expressed mostly by DCs and TAMs, and is also highly expressed by Tregs within tumor draining lymph nodes [10]. The natural role of IDO is to promote tolerance to self-antigens, and within the tumor microenvironment, this negative regulator serves to inhibit local immune activation [11]. Dr. Munn’s work demonstrated that the expression of IDO by stromal cells, which activates the general control non-depressible 2 (GCN2)-kinase-dependent stress response, can directly induce anergy in effector T cells while causing concomitant maturation of functionally quiescent Tregs [12]. This creates a double whammy for tumor immunity. Downstream IDO metabolites can also induce the secretion of pro-tumorigenic growth factors as well [13].

In addition to IDO, the metabolic enzyme arginase (ARG) produced by MSDCs can also promote tumor escape. Dr. Vincenzo Bronte discussed the toleragenic environment in the spleen, where many immature MDSCs are recruited. These CD11b^+^/Gr-1^int^ cells are directed to the spleen by signaling through chemokine receptor type 2 (CCR2 or CD192) and its ligand chemokine ligand 2 (CCL2, also called monocyte chemotactic protein-1 (MCP-1)). Once in the marginal zone of the spleen, these “super-suppressors” induce inactivation of CD8^+^ effector T cells through cross-presentation. Multiple chemotherapy drugs, including 5-Fluorouracil (5-FU) and Gemcitabine, were able to abrogate the negative influence of these MDSCS. Dr. Bronte’s group also showed that MDSCs and CD8^+^ central memory T cells occupy the same niche in the spleen, and that this niche could be emptied for T cells by treating with 5-FU prior to adoptive cell transfer [14].

In his presentation, Dr. Thomas Gajewski showed that about one third of human melanomas are T cell rich [15], with this “inflamed” phenotype driven by type I IFN signaling and CD8α DCs [16], whereas the remainder are T cell poor [15]. This differential phenotype may be determined by tumor variability at the somatic level (driver oncogenes), the genetic level (type I IFN signaling), or the environmental level (exposure to microbes). In preclinical models, Dr. Gajewski showed that tumor DNA taken up by DCs induced the secretion of interferon-β (IFN-β) through interferon regulatory factor-3 (IRF-3) and stimulator of interferon genes (STING)-dependent pathways. This DC-based innate sensing of the tumor resulted in CD8^+^ T cell priming, interferon gamma (IFN-γ) production, and T cell recruitment to the tumor site. Highlighting the complexity of melanoma-specific tumor immunity, a subgroup of melanoma patients with tumors characterized by a similar “inflamed” gene signature and increased T cell infiltration that did not regress developed high levels of expression of the negative regulatory factors PDL-1, IDO, and forkhead box P3 (FoxP3). In addition, some tumor infiltrating CD8^+^ T cells became anergic, with increased expression of early growth factor response gene 2 (Egr2) driving the expression of anergic markers like lymphocyte activation gene-3 (LAG-3) and cytotoxic and regulatory T cell molecule (CRTAM) [17].

Dr. Pierre Coulie’s presentation examined the functional status of T cells in subcutaneous melanoma metastases. This revealed an inflammatory, T helper type 1 gene signature associated with an increase in activated, IFN-γ-producing T cells within the tumor by gene expression profiling and IHC. He also demonstrated that 15-20% of melanoma metastases were accompanied by the formation of active lymphoid aggregates with evidence of both germinal center formation and antibody affinity maturation, indicating the presence of an active adaptive immune response at these sites [18]. Some of these metastases contain T cells and others do not. For some of those that do, there was a lack of cytotoxic T lymphocyte (CTL) killing within the tumor. Thus, TILs may also demonstrate dysfunctional killing capacity that allows the tumor to escape spontaneous rejection by T cells.

In summary, distinct components of the tumor microenvironment can suppress active antitumor T cell responses in multiple ways. Endothelial cells may present a barrier to T cell infiltration, the expression of immunoregulatory molecules such as PDL-1 on tumor cells, IDO by antigen presenting cells, and ARG by MDSCs can blunt the CD8^+^ T cell response, and FoxP3^+^ Tregs can inhibit T cell activity. Finally, dysfunctional or anergic CD8^+^ T cells may demonstrate an intrinsic decrease in killing capacity, in part through increased expression of both LAG-3 and Egr-2.

## Reprogramming the tumor microenvironment to promote tumor regression

Despite the many immunosuppressive mechanisms that blunt productive immune responses, it is clear that the presence of immune cell infiltrates are associated with improved survival and response to therapy in some patients. These observations suggest that there are some inherent differences in the immune response that promote tumor progression in some while promoting tumor regression in others. They also imply that the tumor microenvironment represents a therapeutic target that can be manipulated to promote tumor regression in more patients.

Dr. Samuel Silverstein demonstrated that successful tumor eradication is directly related to the concentration of cytolytically active CD8^+^ T cells that are present in the tumor. He showed that there is a critical concentration of T cells necessary to halt tumor growth. At this critical threshold, the rate of tumor clearance is equal to the rate of tumor growth [19]. Furthermore, effective tumor clearance could only be achieved when the concentration of cytolytically active antigen-specific T cells rises above the critical concentration threshold. He went on to demonstrate that not all intratumoral antigen-specific T cells are cytolytically active, and that exposure to antigen alone increased T cell proliferation without increasing cytolytic activity. Furthermore, an increase in cytolytic activity was only achieved by exposure to antigen in the presence of adjuvant. His studies elegantly demonstrate that it is necessary to consider the concentration, inherent cytolytic capacity, and environmental context of intratumoral T cells in the design of immunotherapies.

One strategy for increasing the presence of cytolytically active, antigen-specific T cells in the tumor is through the adoptive transfer of T cells genetically engineered to force the expression of tumor-antigen specific T cell receptors. Dr. Laurence Cooper shared data demonstrating genetic approaches to produce clinical grade high affinity T cells engineered to express antigen specific surface receptors that allow the recognition of antigen independent of major histocompatibility complex (MHC). This can be achieved by genetically editing CD19-specific chimeric antigen receptor (CAR) T cells to eliminate expression of the endogenous αβ T-cell receptor (TCR) without compromising CAR-dependent effector functions. Genetically modified T cells were generated using the Sleeping Beauty system to stably introduce the CD19-specific chimeric antigen receptor with subsequent permanent deletion of α/β TCR chains with zinc finger nucleases. Preclinical animal studies using these genetically modified T cells have shown success in targeting CD19 on B cells in hematopoietic malignancies [20], and this approach is currently in clinical testing.

The dynamic interplay between immune cells and tumor cells within the tumor microenvironment rests on a multitude of negative immunoregulatory molecules that can be targeted for therapy. Cytokines, such as TGF-β, actively secreted in the tumor microenvironment blunt inflammatory reactions induced by T cells upon TCR engagement, resulting in upregulation of programmed cell death-1 (PD-1) on the T cell surface. PD-1 interacts with tumor-associated PDL-1 to inhibit T cell activity. Dr. Drew Pardoll reported data testing the clinical use of a monoclonal antibody specific for PD-1 to block signals that shut T cells down, thereby promoting T cell-mediated tumor regression [21]. The results were striking, with objective response rates of 18-25% in renal cell carcinoma, melanoma, and non-small cell lung cancer (NSCLC). In addition, many patients had prolonged stable disease lasting over one year. Some patients with high levels of PDL-1 expression at the tumor site had better responses to anti-PD-1 therapy than those with tumors who expressed little to no PDL-1, suggested that PDL-1 could represent a marker that predicts response to therapy. Two additional observations emerged. First, the trafficking of IFN-γ-secreting T cells to the tumor site induces PDL-1 expression by the tumor, resulting in adaptive immune resistance [22]. Additionally, the investigators observed that a few patients with NSCLC previously treated with 5-azacytidine and entinostat had a marked response to subsequent anti-PD-1 therapy. They demonstrated that the epigenetic therapy induced PDL-1 expression at the tumor site in these responding patients, likely through de-methylation of the PDL-1 gene locus. Thus, epigenetic modulation is thought to have rendered the tumors more responsive to anti-PD-1 therapy [23]. Dr. Samir Khlief presented preclinical modeling of combination therapy with cyclophosphamide (CY), a vaccine specific for human papilloma virus (HPV) E7, and anti-PD-1 antibody. This combination therapy resulted in an increase in CD8^+^ T cells in responders. Anti-PD-1 antibody prolonged the inhibition of FoxP3^+^ Tregs by CY, and synergistically decreased the number of FoxP3^+^ Tregs within the spleen and the tumor microenvironment [24]. Similar studies were carried out combining vaccine and CY with B7-DC-Ig (PDL-2). Here, B7-DC-Ig resulted in a significant decrease in the level of PD-1^high^ CD4^+^ T cells (enriched for Tregs) within the tumor. Treatment with B7-DC-Ig also led to a decrease in exhausted PD-1^high^ CD8^+^ T cells, tipping the balance toward functional PD-1^low^ CD8^+^ T cells. Together, these clinical and preclinical studies illustrate the promise of targeting distinct regulatory pathways to enhance the efficacy of current immunotherapies [25].

Cytotoxic T-lymphocyte antigen-4 (CTLA-4) is also a well-established immunologic checkpoint molecule. Dr. Antoni Ribas presented data demonstrating that ipilimumab, a CTLA-4-specific blocking antibody, induces a dense infiltrate of CD8^+^ TILs within the tumor independent of clinical response, where about half the patients with disease progression had immune infiltrates in their tumors. Differences in the quantity, function, or physical location of the T cells did not account for clinical response. Interestingly, analysis of Ki67 expression by TILs and whole body imaging by positron emission tomography (PET) indicated that T cell proliferation in response to therapy occurred in the spleen and not at the tumor site. These data suggest that multiple pathways may require manipulation in order to achieve tumor regression, where one signal promotes the migration of T cells into the tumor, and the second promotes T cell cytotoxicity at the tumor site [26].

An emerging concept is that signaling pathways activated in malignant cells that are essential to support tumor proliferation and survival may act in part through the immune system. Dr. Dean Felsher discussed the concept of “oncogene addiction”, where oncogenes such as MYC are an absolute requirement for tumor growth. Oncogenic proteins that represent the linchpin of tumor growth and progression can be very effectively targeted to induce cellular senescence and promote tumor regression [27]. Notably, Dr. Felsher and his colleagues found that inhibition of oncogene driven cell growth is dependent on the presence of an active immune response, specifically cytokine-secreting CD4^+^ T cells [28].

Cells of the innate immune system may also provide convenient cellular targets to boost the local anti-tumor response. Dr. Phillipe Bousso presented imaging studies using intravital multiphoton microscopy to visualize natural killer (NK) cell dynamics within tumors. NK cells normally recognize stress ligands on tumor cells via natural killer group 2, member D (NKG2D) receptors. Upon recognition of NKG2D ligands, such as Rae-1β, NK cells attach transiently to tumor cells and induce apoptosis, visualized in Dr. Bousso’s studies with the use of fluorescence resonance energy transfer (FRET) combined with differential staining of NK cells [29]. This produces color changes within tumor cells containing activated caspase 3. The interaction between NK cells and tumor cell targets was prolonged with the addition of tumor-specific monoclonal antibodies targeting CD4, which is expressed on the surface of the murine lymphoma cell line EG7. CD4-specific antibodies themselves engaged Fc receptors on NK cells, prolonging cell contact without enhancing degranulation [30].

An imbalance of effector cells to regulatory cells within the tumor microenvironment also plays a key role in cancer persistence in the host. A variety of therapeutic strategies aim to correct this imbalance, thereby redirecting the immune cells in the tumor towards a productive immune response. Dr. Thomas-Oliver Kleen presented a novel PCR-based method for measuring and monitoring changes in the effector T cell:Treg balance through the use of epigenetic immune cell markers. Using this highly sensitive method to analyze 616 samples from breast, ovarian, and colorectal cancers, Dr. Kleen showed that the intrinsic CD3^+^/FoxP3^+^ T cell ratio is unfavorable in a variety of tumor types (unpublished data).

Another imbalance can be found between T helper type 17 (Th17) cells and Tregs. Dr. Weiping Zou discussed the importance of manipulating this balance to favor Th17 cells and promote long-term immunity in an adoptive transfer model using NOD/SCID mice implanted with human ovarian cancer xenografts. Only simultaneous transfer of both Th17 and CD8^+^ T cells produced effective tumor clearance. Gene expression analysis revealed that Th17 cells show increased expression of stem cell genes, such as Notch, and show an increased resistance to apoptosis. It is thought that the persistence of these Th17 cells within the tumor microenvironment is essential for the production of long lasting immunity. Dr. Zou also showed data that pointed to a role for MDSCs in promoting tumor persistence through increasing ovarian cancer cell stemness. His data showed that MDSCs induced the expression of microRNA101, which targets the Notch co-repressor C terminal binding protein 2 (CtBP2). This ultimately resulted in a derepression of CtBP2 target genes to increase the stemness and persistence of ovarian cancer cells [31].

Dr. Nicholas Restifo proposed a potential therapeutic solution for the presence of MDSCs. By modifying T cells to secrete IL-12 and using these T cells in adoptive cell therapy, Dr. Restifo’s lab was able to show enhanced anti-tumor immunity dependent on the cross presentation of tumor antigen and the production of IFN-γ by CD8^+^ T cells in vivo [32]. In these experiments, pmel-1 CD8^+^ T cells, which express a transgenic TCR specific for the melanoma-associated antigen, gp100, were transduced to express high levels of IL-12. Treatment of B16 melanoma tumor bearing mice by adoptive transfer of these cells led to increase in CD8^+^ T cell infiltration. Genetic analysis of the tumors showed an increase in an inflammatory gene signature with a marked increase in genes associated with antigen presentation and processing. Further cellular analysis of the tumors by flow cytometry indicated an increase in the number of CD11b^+^ cells bearing the IL-12 receptor. Additionally, flow sorting followed by real time PCR (rtPCR) of these CD11b^+^ myeloid cells indicated programmatic changes indicative of an increased inflammatory response through an increase in IFN-γ. Functional analysis of the myeloid cells from the tumor showed a marked increase in their ability to stimulate T cell proliferation [33]. These experiments demonstrate that tumor-suppressive mechanisms can be overturned to support potent antitumor immunity, where inflammation driven by IFN-γ and its targets reprograms the immune cells within the tumor microenvironment to successfully induce tumor regression.

When designing effective immunotherapies, it may be equally important to consider not only the tumor microenvironment before and after vaccination, but also to consider the microenvironment of immune priming at the vaccine site itself. Whole cell granulocyte-macrophage colony-stimulating factor (GM-CSF)-secreting vaccines (GVAX) have been used in pre-clinical modeling and clinical trials to treat a variety of both solid and hematologic malignancies. Dr. Glen Dranoff described his findings elucidating the dual role of GM-CSF in immune protection and immune tolerance. The context in which antigen presenting cells were exposed to GM-CSF at the site of immune priming greatly influenced the resulting immune response within the tumor microenvironment. Initial in vitro studies showed when GM-CSF-proficient APCs encountered apoptotic cells in the presence of milk fat globule-EGF factor-8 protein (MFG-E8, or lactadherin), the secretion of TGF-β and CCL22 was increased compared to GM-CSF-deficient APCs. In vivo, these factors support the recruitment of Tregs, blunting the immune response. Translating these studies in vivo using a therapy model with B16 mice, when a GM-CSF vaccine was engineered to express a dominant negative mutant of MFG-E8 (RGE), vaccine efficacy was restored and tumor-free survival was increased. Abrogating the influence of MFG-E8 enhanced the phagocytic capacity of CD11b^+^ myeloid cells, thus increasing both the CD8^+^/ FoxP3^+^ T effector cell ratio and humoral immunity [34]. In addition, MFG-E8 blockade in combination with chemotherapy, molecularly targeted therapy, and radiation induced the destruction of various tumor types in preclinical models. These combination treatments provoke extensive tumor cell apoptosis, leading to highly effective cross-presentation of dying tumor cell to DCs [35].

Dr. Dranoff also discussed an innovative approach to intensifying tumor immunity through improving the in vivo delivery of immunomodulating agents at the time of immune priming. Porous polymer scaffolds containing GM-CSF, the toll-like receptor (TLR) agonist CpG, and tumor cell lysates were implanted subcutaneously in mice. These “immunologically charged” scaffolds enhanced DC maturation, decreased suppressive cytokines such as IL-10 and TGFβ, and increased Th1-cytokines. This novel polymer-based delivery prolongs immune priming through slow metabolism of the polymer into lactic acid with the gradual, steady release of its contents, thereby creating long-lasting anti-tumor immunity. Thus, engineered scaffolds might provide a means to enhance tumor selectivity, both promoting a durable immune response while simultaneously reducing the risk of inflammatory toxicity [36].

Tumor specific immune responses may be measured directly at the tumor site, or indirectly within the peripheral blood. Dr. Mary Disis reported the presence of autoimmune serologic response signatures that may be predictive of therapeutic response to vaccination in breast cancer. Retrospective data analysis of phase 1 and phase 2 clinical trials using HER-2 peptide vaccine given with adjuvant GM-CSF demonstrated an association between epitope spreading and survival [37,38]. Serological analysis was performed using serological analysis of recombinant tumor cDNA expression libraries (SEREX) comparing antibody response signatures from long term survivors who underwent epitope spreading to non-responders. Here, an autoantibody signature predicted survival. Peripheral blood was also analyzed by human GeneChip microarray, revealing a type 1 IFN gene signature detectable within the peripheral blood of responders (unpublished data). Dr. Disis went on to hypothesize that the development of autoantibodies to nucleosome-, DNA-, or RNA bound proteins may increase the uptake of these proteins by DCs. When released inside the cell, they may propagate a type 1 interferon response through toll-like receptor (TLR) signaling, thereby augmenting cross-priming and epitope spreading. Future studies aim to further characterize these immune response signatures in order to define a signature of immune-mediated tumor rejection that is independent of the therapeutic modality.

Profiling the cytokine milieu within the tumor microenvironment may also predict disease outcome. Dr. Helen K. Angell showed that interleukin-15 (IL-15) is an important predictor of the metastatic potential of primary colorectal cancers after surgery. The presence of deletions in the IL-15 gene in patients strongly correlated with increased metastasis, whereas high IL-15 expression correlated with increased disease free survival. Additionally, the presence of high levels of IL-15 were correlated with increased numbers of Ki67^+^ CD3^+^ T cells and CD20^+^ B cells in patients with longer survival. Digital imaging analysis revealed proliferating cells mostly within the invasive margin of the tumors (unpublished data).

## Clinical strategies for integrative immunotherapies: inciting lasting immunity in the tumor microenvironment

Preclinical studies elucidating the dynamic host-tumor cell interactions within the microenvironment have laid a solid foundation for the development of novel immunotherapies that integrate agents specific for immunoregulatory pathways with agents that generate increased numbers of antigen-specific T and B cells. The first clinical interventions that aim to target the microenvironment to enhance tumor immunity are under active evaluation in the clinic.

Dr. Brian Rini discussed the use of the antiangiogenic molecule sunitinib for the treatment of renal cell carcinoma (RCC). In addition to antiangiogenic activity, sunitinib can decrease splenic MDSCs and increase intratumoral CD3^+^ IFN-γ-secreting T cells in preclinical models. In metastatic RCC patients, sunitinib reduced both MDSC and Tregs in the peripheral blood, and increased IFN-γ secretion by T cells [39]. These observations led to the design of a trial to define how sunitinib might alter the tumor microenvironment in RCC patients. In this trial, neoadjuvant sunitinib treatment resulted in a decrease in CD33^+^HLADR^-^ MDSCs and a concomitant increase in CD3^+^ IFN-γ-secreting T cells in a small subset of patients. Patients with no notable changes in MDSC number displayed upregulation of the pro-angiogenic matrix metalloproteinases MMP8 and MMP9, and interleukin 8 (IL-8) (unpublished data). The effect of these distinct tumor responses to sunitinib treatment on patient outcome or acquired drug resistance to sunitinib is not yet known. Future studies will combine sunitinib with cyclophosphamide and IMA901, a vaccine containing multiple RCC associated peptides. Early data shows that lower baseline MSDC levels are associated with longer overall survival in these patients [40].

Dr. Dimitry Gabrilovich discussed regulation of the immune response through the inhibition of MDSCs. He has demonstrated that all-trans retinoic acid (ATRA) can reduce the number of CD11b^+^CD14^+^CD33^+^ cells in small cell lung carcinoma (SCLC) patients. He hypothesized that removing suppressive myeloid populations could enhance the efficacy of vaccination with p53-specific peptide vaccines, and reported a clinical study testing this concept. Early results revealed that combination therapy increased both p53-specific immune responses and the number of granzyme B^+^ CD8^+^ T cells in the blood (unpublished data). Further analyses will determine if this combination immunotherapy will provide an overall clinical benefit to patients suffering from SCLC.

Dr. Ivan Borrello demonstrated a novel activity for phosphodiesterase type 5 (PDE5) inhibitors in decreasing the production of inducible nitric oxide (iNOS), arginase, and IL-4Rα by MDSCs [41]. In a neoadjuvant trial, he showed that Tadalafil, a PDE5 inhibitor, could effectively target MSDCs and potentiate immune responses in patients with head and neck squamous cell carcinoma (HNSCC) 2 weeks prior to surgery or radiation therapy. Patients receiving the PDE5 inhibitor showed a significant increase in both candida-specific delayed type hypersensitivity (DTH) responses and T cell expansion to CD3 plus CD28 stimulation, and a decrease in MDSC compared to the patients receiving placebo. These data reveal a novel immunoregulatory activity associated with PDE5 inhibition. There also appeared to be an enhanced number of CD69^+^CD4^+^ T cells with no corresponding increase in CD8^+^ T cells. Lastly, PDE5 inhibition enhanced tumor-specific T cell responses when patient-derived DCs were pulsed with tumor lysate and co-cultured with patient T cells (unpublished data). These data therefore demonstrated the ability of PDE5 to augment the global immune response in HNSCC by decreasing MDSCs and increasing T cell proliferation. Future studies aim to examine the combination of a PDE5 inhibitor with chemotherapy and vaccine to determine the efficacy in enhancing the immune response through modulation of MDSCs.

Combination immunotherapy trials designed to test vaccines in sequence with cyclophosphamide (CY) to mitigate the influence of Tregs, or in combination with CY and a therapeutic monoclonal antibody (Trastuzumab (Herceptin)), to enhance antigen processing and presentation were discussed by Dr. Leisha Emens. Here, a HER2-expressing whole cell GM-CSF secreting breast tumor vaccine was tested in sequence with a range of low, immune modulating doses of CY and doxorubicin (DOX) in patients with metastatic breast cancer. This study demonstrated that 200mg/m^2^ CY and 35mg/m^2^ DOX were able to maximally induce HER2-specific antibody responses, while maintaining the HER-2-specific DTH response. The optimal dose of CY tested was able to decrease peripheral Tregs without decreasing effector T cell populations [42]. In a second trial, low dose CY (300 mg/m^2^) was sequenced with vaccine in the setting of standard weekly trastuzumab therapy for HER-2^+^ metastatic breast cancer. Here, about 50% of patients showed a clinical benefit at 6 months following treatment, and about 35% showed a clinical benefit at 1 year following treatment (unpublished data). This multi-agent immunotherapy is now being tested for use in HER2-negative patients, where a single dose of Trastuzumab is given with CY at the time of immune priming. These studies aim to determine optimal immunotherapeutic treatment strategies to enhance the immune response and promote tumor regression in distinct breast cancer subtypes.

## The immunoscore

While promising immunotherapies are being tested in clinical trials, it remains unclear how to select the patients most likely to respond. A hurdle in predicting potential responders to immunotherapy is that current tumor staging does not take the immune context of the tumor into account. Incorporating immune cell analyses into the standard staging based on tumor size, lymph node status, and metastatic disease (TNM stage) may better select those patients who may benefit from immunotherapy as well as improve patient stratification and prognosis for standard cancer therapy.

Dr. Alessandro Lugli discussed the importance of immune cells as prognostic markers in colorectal cancer (CRC). He showed that CD8 and receptor for hyaluronan-mediated motility (RHAMM or CD168) are important biomarkers in rectal cancer, where lack of CD8^+^ TIL and high levels of RHAMM led to lower survival independent of disease stage [43]. In particular, patients with T1 and T2 tumors that express high levels of RHAMM but contain no T cells have a 5-year cancer-specific survival rate similar to patients with T3 or T4 cancers. In CRC CD8^+^CD45RO^+^ effector memory cells are a valuable predictor of favorable outcome in early stage colon cancer [44]. Furthermore, the absence of CD8^+^ T cells predicts shorter survival in stage II colon cancer patients. In particular, urokinase-type plasminogen activator (uPA) positive, T3 tumors without CD8^+^ cells showed no difference in survival compared to T4 patients [45]. This is especially significant for stage II CRC patients because currently they are not recommended for adjuvant therapy, and, these data imply that T3 patients with CD8^-^ uPA^+^ lymph node negative tumors may derive a survival benefit from adjuvant therapy. These data illustrate the potential value of immune cells as predictors of disease-free survival, and possibly therapeutic response, in CRC.

Dr. Jerome Galon has led a major effort to promote greater understanding of the importance of TILs as a powerful predictor of patient survival. He and his colleagues have shown that the type, density, and location of immune cells within the tumor microenvironment of CRC have prognostic value that is superior to and independent of the TNM classification. The presence of CD8^+^ T cells in particular are powerful predictors of decreased local recurrence, and improved disease-free survival and overall survival in patients with CRC, as demonstrated in a large multivariate COX proportional hazard analysis of 499 patients with Stage I, II, and III CRC. These studies resulting in a proposed Immunoscore based on CD3 and CD8 staining within the tumor and at the invasive margin of CRC [46,47].

Developing this further, Dr. Galon is on the SITC Taskforce steering committee that aims to validate the Immunoscore and promote its use as one of the standard histopathological assessments at the time of cancer diagnosis. Specifically, the Taskforce aims to validate the Immunoscore as an independent prognostic marker for CRC and potentially as a new classification of CRC. The task force has organized 22 centers in 16 countries to undertake this validation, with a centrally managed data cloud that will allow participating centers to share and store digital images [48]. Current studies are also underway evaluating whether the CD3 and CD8 Immunoscore or some other combination of immune markers may serve as a prognostic marker in at least 18 other cancers. Finally, studies are underway to investigate whether a blood-based Immunoscore may be of value for situations where tissue is inaccessible or not available.

The Immunoscore provides a way for clinicians to have a “sneak peek” at the immune status within tumor microenvironment to help determine treatment strategies. Determining individual patient’s Immunoscores may allow clinicians to better predict survival and therapeutic response. Dr. Bernard Fox emphasized the importance of implementing the Immunoscore to facilitate the stratification of patients into potential responders (T cell-rich) and nonresponders (T cell-poor) to currently available immunotherapies, and possibly other therapies as well. This invaluable information may allow T cell-poor patients to be treated first by therapies that will stimulate an immune response at the tumor site. If that is not possible, it may indicate a need for the development of a second generation of immunotherapeutics that are more capable of igniting the immune response via new, improved vaccination strategies and/or multiple immune checkpoint blockade strategies to overcome tumor resistance and increase the efficacy of immunotherapies in these patients.

Dr. Francesco Marincola concluded the workshop by discussing the immune-mediated destruction of tumors, and highlighted the concept of prognostic, predictive, and mechanistic immune signatures along the continuum of immune surveillance. He emphasized the need for the application of multivariate analyses to adequately powered clinical data sets to assess immune responsiveness, and how it is influenced by the host genetic background as well as tumor intrinsic and environmental factors. He reviewed data showing that genetic signatures can predict melanoma tumor regression post-immunotherapy, and that a genetic mutation in CCR5 at the host level, specifically the delta-32 mutation, is associated with shorter disease-specific survival [49]. In melanoma, only when delta-32 mutations are considered and both CCR5 expression and CXCR3 expression are analyzed can these genes significantly predict complete responders, indicated by an odds ratio of greater than 6 [50].

## Conclusions

The research presented at the 2012 SITC Workshop highlighted a rapidly growing appreciation for the complexities of the tumor microenvironment, and how they might be manipulated therapeutically to promote or even provoke effective tumor immunity. Importantly, accurately profiling the immunobiology of the tumor microenvironment—and standardizing an Immunoscore—will better define prognosis and response to both immune-based and standard cancer therapies. The future of immunotherapy will almost certainly involve integrative strategies that target multiple immune modulating networks and effector cells to not only to amplify existing immune responses in patients predicted to be immunologic responders, but also to stimulate new immunity that can be subsequently amplified in patients predicted to be immunologic nonresponders. Using integrated, sequential combination treatment strategies matched to both the genetics and immunobiology of a patient’s tumor will make personalized immunotherapy a reality, leading to increased cancer cure rates and ultimately effective cancer prevention strategies.

## Abbreviations

SITC: Society for Immunotherapy of Cancer; TILs: Tumor infiltrating T lymphocytes; TECs: Tumor endothelial cells; IHC: Immuohistochemistry; ETBR: Endothelin type B receptor; ICAM-1: Intercellular Adhesion Molecule 1; FasL: Fas-ligand; IL-10: Interleukin-10; TGF-β: Transforming growth factor-beta; PDL-1: Programmed cell death ligand-1; PDL-2: Programmed cell death ligand-2; IDO: Indoleamine 2,3-dioxygenase; ARG: Arginase; iNOS: Inducible nitric-oxide synthase; DCs: Dendritic cells; MDSCs: Myeloid-derived suppressor cells; TAMs: Tumor-associated macrophages; Tregs: T regulatory cells; IFN-γ: Interferon gamma; GCN2: General control non-depressible kinase 2; CCR2: Chemokine receptor type 2; CCL2: Ligand chemokine ligand 2; MCP-1: Monocyte chemotactic protein-1; IFN-β: Interferon-beta; IRF3: Interferon regulatory factor-3; STING: Stimulator of interferon genes; EGFR: Epidermal growth factor receptor; LAG-3: Lymphocyte activation gene-3; CRTAM: Cytotoxic and regulatory T cell molecule; CTL: Cytotoxic T lymphocyte; FoxP3: Forkhead box P3; MHC: Major histocompatibility complex; CARs: Chimeric antigen receptors; TCR: T-cell receptor; CY: Cyclophosphamide; HPV: Human papilloma virus; CTLA-4: Cytotoxic T-lymphocyte antigen-4; PET: Positron emission tomography; NK: Natural killer cell; NKG2D: Natural killer group 2, member D receptors; FRET: Fluorescence resonance energy transfer; Th17: T helper type 17 cells; CtBP2: C terminal binding protein 2 (.; IL-12: Interleukin 12; GM-CSF: Granulocyte-macrophage colony-stimulating factor; GVAX: Whole cell granulocyte-macrophage colony-stimulating factor- secreting vaccines; CCl22: Chemokine ligand 22; APC: Antigen presenting cell; MFG-E8: Milk fat globule-EGF factor-8 protein; TLR: Toll-like receptor; Th1: T helper type 1; SEREX: Serological analysis of recombinant tumor cDNA expression libraries; IL-15: Interleukin-15; MMP: Matrix metalloproteinases; IL-8: Interleukin 8; SCLC: Small cell lung carcinoma; ATRA: All-trans retinoic acid; PDE5: Phosphodiesterase type 5; DTH: Delayed type hypersensitivity; HNSCC: Head and neck squamous cell carcinoma; IL-4Rα: Interleukin receptor 4alphaof; iNOS: inducible nitric oxide; DOX: Doxorubicin; RHAMM: Receptor for hyaluronan-mediated motility; CRC: Colorectal cancer; uPA: urokinase-type plasminogen activator; AJCC/UICC: American Joint Cancer Committee/Union Internationale Contre le Cancer; ICR: Immunologic constant of rejection; CXCR3: Chemokine receptor 3; CCR5: Chemokine receptor 5.
